# Prevalence of vitamin D deficiency among children with sepsis, its association with sepsis severity and its outcome in a pediatric ICU

**DOI:** 10.1186/cc12935

**Published:** 2013-11-05

**Authors:** Ponnarmeni Satheesh, Savita Verma, Sunit Singhi, Arun Bansal

**Affiliations:** 1 Department of Paediatrics, Post-Graduate Institute of Medical Education & Research (PGIMER), Chandigarh, India

## Background

Increased prevalence of vitamin D deficiency (VDD) in sepsis and its association with sepsis severity has been documented in adults [1-3]. However, data on the pediatric population are scarce. This study aims at assessing the prevalence of VDD (25-hydroxyvitamin D (25(OH)D) level <20 ng/ml) among children with sepsis in developing nations and its implication on sepsis severity.

## Materials and methods

A prospective observational study conducted between January and December 2012. During the study period all consecutive PICU admissions between the ages of 1 and 12 years were screened for sepsis at the time of admission to the ICU. Out of 613 PICU admissions, 124 patients satisfying the criteria for sepsis [4] were randomly enrolled and followed up throughout the hospital stay. Patients with an immunosuppressed state or receipt of vitamin D within the 3 months prior to hospital admission were excluded. A control group comprising of 40 healthy children was also included for comparison with the general population. The serum 25(OH)D level was measured in all patients with sepsis within 24 hours of admission to the PICU. Severity of sepsis was assessed using the Pediatric Risk of Mortality III (PRISM III) score and the daily Sequential Organ Function Assessment (SOFA) score.

## Results

Patients with sepsis had low 25(OH)D levels compared with healthy controls (*P *= 0.04). Median 25(OH)D level among patients was 19.7 ng/ml (interquartile range (IQR): 12.5, 31.2) and median 25(OH)D level among controls was 30.4 ng/ml (IQR: 22.1, 38). Prevalence of VDD was high among patients 51% (95% confidence interval (CI), 42 to 59) compared with the VDD of 17% (95% CI, 8 to 32) in healthy controls (*P *< 0.001) (Table 1). No significant correlation was found between vitamin D level and PRISM III score or daily SOFA score. Out of 19 deaths, 17 (90%) deaths occurred in patients with vitamin D deficiency and insufficiency (odds ratio 3.09, 95% CI: 0.6 to 20.7). However, the difference in mortality was not statistically significant (*P *= 0.58). Factors such as septic shock, multiorgan dysfunction syndrome (MODS), duration of mechanical ventilation, blood culture positivity, hypocalcemia and length of PICU stay were not modified by the presence of VDD (Table 2).

**Table 1:** Vitamin D status among patients with sepsis and healthy controls

	**Vitamin D status, % (*n*)**		
	
	**Deficient, 25(OH)D <20 ng/ml**	**Insufficient, 25(OH)D = 20 to 30 ng/ml**	**Sufficient, 25(OH)D >30 ng/ml**
	
Patients (*n *= 124)	51 (63)	25 (31)	24 (30)
Controls (*n *= 40)	18 (7)	30 (12)	52 (21)

*P *< 0.001.

**Table 2:** Comparison of clinical characteristics of patients with sepsis by vitamin D status

	Vitamin D deficient (*n *= 63)	Not deficient (*n *= 61)	*P *value
Age (years)	4.2 (2.1 to 9.4)	4.2 (1.8 to 8.5)	0.60
Weight (kg)	15 (10.3 to 24.5)	14 (10 to 20)	0.67
PRISM III score	17 (13 to 22)	14 (13 to 22)	0.26
Mean SOFA score from day 1 to day 5	4 (2 to 6.8)	4 (2 to 6.3)	0.64
Septic shock	52 (32)	49 (30)	0.85
MODS	58 (35)	56 (34)	0.83
Mechanical ventilation (hours)	96 (24 to 144)	120 (72 to 216)	0.52
Bacterial culture positivity	23 (15)	34 (21)	0.19
PICU stay (hours)	86 (12 to 114)	90 (43 to 168)	0.73
Hypocalcaemia (calcium <8 mg/dl)	26 (17)	18 (11)	0.16
Mortality	16 (10)	15 (9)	0.53

Data presented as median (IQR) or % (*n*).

## Conclusions

We found a high prevalence of VDD among children with sepsis when compared with healthy children but VDD was not associated with the severity of sepsis or its outcome.
